# Burden dynamics of pulmonary arterial hypertension in China and the G20 aggregate: a comparative analysis based on the global burden of disease study 2023

**DOI:** 10.1080/07853890.2026.2727194

**Published:** 2026-09-06

**Authors:** Qizhi Wang, Ling Hao, Weitao Cao, Chengyi Hu, Yanze Shu, Wenzhen Liu, Haoyang Hu, Zhuxiang Zhao

**Affiliations:** ^a^ Department of Respiratory and Critical Care Medicine, Guangzhou First People’s Hospital, The Second Affiliated Hospital, School of Medicine, South China University of Technology, Guangzhou, Guangdong, China; ^b^ Guangzhou Medical University, Guangzhou, Guangdong, China; ^c^ Medical School of Chinese PLA, Beijing, China

**Keywords:** ARIMA model, China, G20 aggregate, GBD 2023, population aging, pulmonary arterial hypertension

## Abstract

**Background:**

Pulmonary arterial hypertension (PAH) causes substantial premature mortality and disability. Updated evidence comparing long-term PAH burden in China with the Group of Twenty (G20) aggregate remains limited. We assessed changes from 1990 to 2023 and projected future trends.

**Methods:**

PAH estimates for China and the G20 aggregate were extracted from the Global Burden of Disease Study 2023. Outcomes included incidence, prevalence, deaths, disability-adjusted life years (DALYs), years lived with disability (YLDs), and years of life lost (YLLs), expressed as absolute counts and age-standardized rates. Temporal trends were assessed using Joinpoint regression. Sex-specific and age-specific patterns and demographic decomposition were examined. Autoregressive integrated moving average (ARIMA) models projected overall burden through 2050. Bayesian age-period-cohort (BAPC) models projected sex-specific patterns through 2038.

**Results:**

From 1990 to 2023, incident and prevalent cases and YLDs increased in both settings, whereas deaths, DALYs, and YLLs declined. In China, prevalent cases increased by 63.5%, from 17,020.87 to 27,834.41, while the age-standardized death rate decreased by 74.3%, from 0.29 to 0.08 per 100,000 population. Similar declines in mortality-related age-standardized indicators occurred in the G20 aggregate. Females generally had higher age-standardized prevalence and YLD rates than males, although sex differences in mortality-related outcomes varied by setting. Absolute burden shifted toward older age groups. Population growth and aging contributed to increases in absolute burden, whereas epidemiological change generally acted in the opposite direction. Projections suggested continued declines in age-standardized mortality-related burden through 2050, alongside increases in incident and prevalent case numbers.

**Conclusion:**

Mortality-related PAH burden declined in China and the G20 aggregate, while absolute case numbers increased. Population growth and aging contributed to this divergence, underscoring the need for continued surveillance and long-term health-service planning.

## PLAIN LANGUAGE SUMMARY

- Pulmonary arterial hypertension is a serious disease affecting the blood vessels of the lungs and can lead to right heart failure and premature death.
- We used data from the Global Burden of Disease Study 2023 to compare changes in PAH burden in China and the G20 aggregate from 1990 to 2023 and to project trends through 2050.
- In China, the number of people living with PAH increased by approximately 64%, while the age-standardized death rate decreased by approximately 74%.
- Females generally had higher prevalence and rates of disability than males, although sex differences in death-related outcomes were not consistent between China and the G20 aggregate.
- The absolute burden increasingly shifted toward older age groups. Population aging and population growth contributed to the increasing number of people affected.
- Future declines in death-related burden may therefore occur alongside continued growth in the number of people living with PAH, with implications for disease surveillance and long-term care planning.

## Introduction

Pulmonary arterial hypertension (PAH) is a progressive and life-threatening pulmonary vascular disease characterized by increased pulmonary vascular resistance and pulmonary arterial pressure, which may ultimately lead to right ventricular failure and premature death [1]. Although advances in diagnostic approaches and PAH-targeted therapies have improved clinical outcomes, PAH continues to impose a substantial burden because of its high morbidity, premature mortality, impaired quality of life, and considerable healthcare costs [2,3]. From a population-health perspective, accurately characterizing the magnitude and temporal evolution of the PAH burden is important for disease surveillance, clinical management, and healthcare resource allocation.

The Global Burden of Disease (GBD) Study provides a comprehensive and standardized framework for quantifying disease burden across countries and over time, enabling robust comparisons of epidemiological patterns across populations [4–6]. Within this framework, the Group of Twenty (G20) constitutes an important population-level reference, accounting for approximately two-thirds of the global population and the majority of global economic output. The G20 aggregate therefore provides a broad benchmark against which the magnitude and temporal trends of the PAH burden in China can be evaluated. Because the G20 estimates are reported as a supranational aggregate, however, they do not represent the burden or temporal patterns of individual G20 members.

Previous studies have described the epidemiological burden and characteristics of PAH across different populations [7–9]. However, evidence comparing China with the G20 aggregate remains limited. In addition, most existing studies were based on earlier GBD releases and did not comprehensively evaluate long-term temporal trends, age- and sex-specific patterns, demographic changes, and future projections. Few studies have used the recently released GBD 2023 estimates to provide an updated comparative assessment of PAH burden in China and the G20 aggregate.

Therefore, we used data from the GBD 2023 Study to compare the burden of PAH between China and the G20 aggregate. Our analysis, covering the period from 1990 to 2023, measured the burden of PAH using absolute numbers and age-standardized rates. We further examined age- and sex-specific patterns, quantified the contributions of population growth, population aging, and epidemiological change, and projected future burden using ARIMA models through 2050 and BAPC models through 2038.

Our findings may provide updated evidence to support PAH surveillance and long-term healthcare planning in China and to contextualize the observed trends relative to the G20 aggregate.

## Methods

### Study design and data source

This study was a population-based comparative analysis using data from the GBD 2023, coordinated by the Institute for Health Metrics and Evaluation (IHME). The GBD database provides comprehensive and standardized estimates of disease burden across 204 countries and territories from 1990 to 2023, including incidence, prevalence, mortality, years lived with disability (YLDs), years of life lost (YLLs), and disability-adjusted life years (DALYs). Detailed methodological descriptions of the GBD study have been published elsewhere.

We extracted estimates for China and the G20 aggregate directly from the GBD Results Tool. The G20 estimates were supranational aggregate estimates provided by IHME and were not calculated by summing or averaging estimates for individual G20 members. Therefore, all comparative analyses in this study were conducted between China and the G20 aggregate rather than among individual G20 countries.

### Case definition

In the GBD 2023 cause hierarchy, the disease examined in this study was defined as ‘Pulmonary Arterial Hypertension’ (cause ID 494). Accordingly, the precise disease term Pulmonary Arterial Hypertension (PAH) has been standardized and applied consistently across all sections of this manuscript. We extracted the estimates assigned to this cause directly from the GBD Results Tool and did not construct a composite PH category or combine estimates across different PH groups. Case ascertainment and cause-specific estimation were based on the data sources and modelling procedures adopted by the GBD 2023 Study. The GBD-labelled PAH category should not be assumed to correspond perfectly to hemodynamically confirmed WHO Group 1 PAH in contemporary clinical practice.

### Outcome measures

The outcomes included incidence, prevalence, deaths, DALYs, YLDs, and YLLs. For each outcome, we extracted absolute numbers and rates, as applicable. Age-standardized rates included the ASIR, ASPR, ASDR, age-standardized DALY rate, age-standardized YLD rate, and age-standardized YLL rate and were expressed per 100,000 population using the GBD standard population. Estimates were presented with their corresponding 95% UIs provided by the GBD 2023 Study.

DALYs represent the sum of YLLs and YLDs. Within the GBD framework, YLLs are calculated using the number of deaths and the standard remaining life expectancy at the age of death, whereas YLDs are estimated from the prevalence of nonfatal health states and their corresponding disability weights, with adjustment for comorbidity.

### Data extraction and stratification

Data were extracted by location, year, sex, age group, measure, metric, and estimate type. The locations included China and the G20 aggregate, and the study period covered 1990–2023. Sex-specific analyses were conducted for females, males, and both sexes, where applicable, and age-specific analyses used the age groups available in the downloaded GBD dataset. The principal comparison was between China and the G20 aggregate.

### Trend analysis

Temporal trends in the burden of pulmonary arterial hypertension (PAH) were assessed using Joinpoint regression analysis. Age-standardized rates (ASRs), including incidence, prevalence, mortality, disability-adjusted life years (DALYs), years lived with disability (YLDs), and years of life lost (YLLs), were analyzed to characterize long-term temporal changes from 1990 to 2023. Details of the Joinpoint regression procedure are described below.

### Joinpoint regression analysis

To further characterize changes in temporal trends during the study period, Joinpoint regression analysis was performed for the annual ASRs of incidence, prevalence, deaths, DALYs, YLDs, and YLLs. The analysis fitted a series of connected log-linear segments and identified joinpoints at which the slope of the temporal trend changed. The annual percentage change (APC) and its 95% CI were estimated for each segment, while the average annual percentage change (AAPC) and its 95% CI were calculated to summarize the overall trend from 1990 to 2023. An APC or AAPC was considered significantly increasing when both the estimate and the lower bound of its 95% CI were greater than 0 and significantly decreasing when both the estimate and the upper bound of its 95% CI were less than 0. When the 95% CI included 0, the trend was considered statistically nonsignificant. Joinpoint analyses were conducted separately for China and the G20 aggregate and were stratified by sex. All analyses were performed using the Joinpoint Regression Program, version 5.0.1.

### Demographic decomposition analysis

To quantify the factors contributing to changes in the absolute burden of PAH between 1990 and 2023, we performed a demographic decomposition analysis based on the Kitagawa–Das Gupta method. For each burden measure, the absolute number was expressed as the sum across age groups of the product of total population size, the proportion of the population in each age group, and the corresponding age-specific rate. The total change between 1990 and 2023 was decomposed into three components: population growth, population aging, and epidemiological change.

The population-growth component represented the change attributable to variation in total population size. The population-aging component represented the change attributable to shifts in the population age structure. The epidemiological-change component represented the change attributable to variation in age-specific rates after accounting for population size and age structure. The contribution of each component was estimated using the symmetric Kitagawa–Das Gupta decomposition formula, which averages the relevant combinations of baseline and final-year population size, age structure, and age-specific rates. Positive values indicated that a component contributed to an increase in absolute burden, whereas negative values indicated that it contributed to a decrease. The epidemiological-change component was interpreted as a residual age-specific-rate component and not as a direct measure of treatment effects, diagnostic improvement, or other specific clinical interventions.

The decomposition was additive, such that the total observed change equaled the algebraic sum of the population-growth, population-aging, and epidemiological-change components. For graphical presentation, the percentage contribution of each component was calculated by dividing its signed absolute contribution by the net observed change and multiplying by 100. Because components may act in opposite directions, their absolute percentage magnitudes may exceed 100%, particularly when the net observed change is small.

### Autoregressive integrated moving average forecasting

Annual PAH burden estimates from 1990 to 2023 were used to fit separate non-seasonal autoregressive integrated moving average (ARIMA) models according to location, sex, burden measure, and metric. Candidate model orders were evaluated using the corrected Akaike information criterion AIC_c_, and the model with the lowest AIC_c_ was selected using an exhaustive, non-stepwise search with exact likelihood estimation. Forecast distributions for 2024–2050 were generated using 1,000 bootstrap simulations. The 2.5th and 97.5th percentiles of the simulated forecast distributions were used to construct 95% prediction intervals. These projections represent statistical extrapolations of historical patterns and should be interpreted as indicative trend scenarios rather than deterministic estimates of future PAH burden.

### Bayesian age–period–cohort analysis

To project sex-specific temporal patterns in PAH burden, Bayesian age–period–cohort (BAPC) models were fitted separately for China and the G20 aggregate. For each location and sex, age-specific outcome counts and the corresponding population denominators from 1990 to 2023 were organized into age-by-period matrices. The models were estimated using the BAPC package (version 0.0.36) and the INLA package (version 24.11.25) in R version 4.4.1.

A Poisson likelihood was assumed for the age-specific counts, with the logarithm of the corresponding population used as an offset. Age, period, and cohort effects were modelled using first-order random-walk (RW1) priors, while the precision parameters followed the default weakly informative log-Gamma priors implemented in INLA. Age-standardized projections through 2038 were obtained using the GBD standard population weights. Uncertainty was summarized using 95% Bayesian credible intervals, defined by the 2.5th and 97.5th percentiles of the posterior predictive distribution. The projection horizon was limited to 2038 because uncertainty increases substantially with longer extrapolation beyond the observed period.

### Statistical software

Statistical analyses were performed using R version 4.4.1. ARIMA forecasting was conducted using the forecast package version 8.23.0, and Bayesian age–period–cohort analyses were performed using the BAPC package version 0.0.36 and the INLA package version 24.11.25. Joinpoint regression analyses were conducted using the Joinpoint Regression Program version 5.0.1.

## Results

### Temporal trends in the burden of pulmonary arterial hypertension, 1990–2023

**Figure 1.**
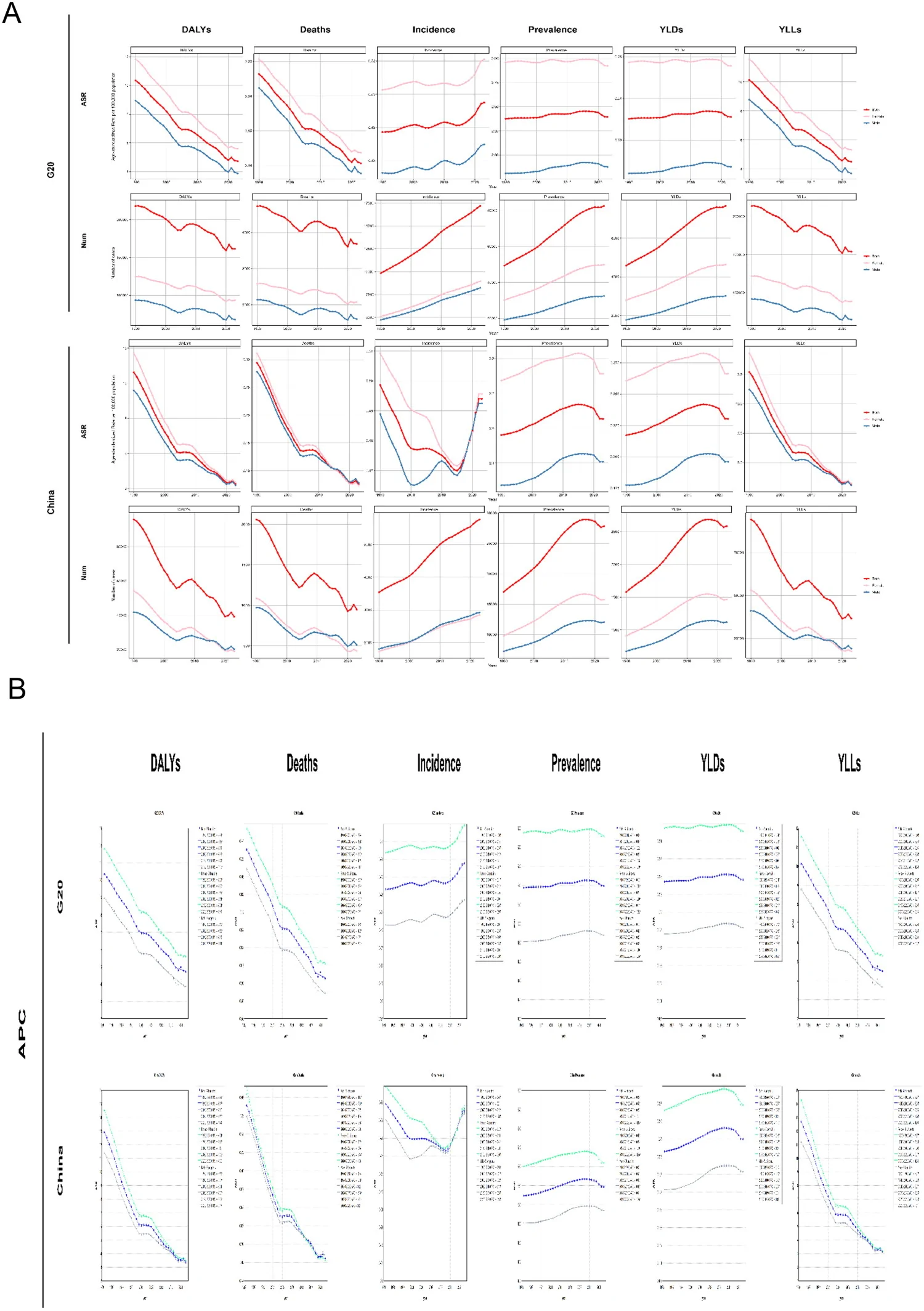
Temporal trends in the burden of pulmonary arterial hypertension in China and the G20 aggregate, 1990–2023. (A) Absolute numbers and age-standardized rates of DALYs, deaths, incidence, prevalence, YLDs, and YLLs. Solid lines indicate point estimates, and shaded areas indicate 95% uncertainty intervals. (B) Joinpoint regression of age-standardized rates, showing annual percentage changes across temporal segments.

From 1990 to 2023, the absolute numbers of incident and prevalent PAH cases and YLDs increased in both China and the G20 aggregate, whereas the numbers of deaths, DALYs, and YLLs decreased (Figure 1A). In China, the number of prevalent cases increased by 63.5%, from 17,020.87 (95% UI, 11,203.63–24,789.09) in 1990 to 27,834.41 (95% UI, 18,527.24–40,429.70) in 2023. The number of prevalent cases in the G20 aggregate increased by 67.3%, from 49,361.77 (95% UI, 33,265.41–71,066.86) to 82,559.16 (95% UI, 55,585.93–119,005.97).

In contrast, mortality-related outcomes declined over the study period. In China, the number of deaths decreased by 53.9%, from 2,060.66 (95% UI, 1,241.26–3,282.42) in 1990 to 949.23 (95% UI, 549.45–1,457.84) in 2023. The age-standardized death rate decreased by 74.3%, from 0.29 (95% UI, 0.18–0.47) to 0.08 (95% UI, 0.04–0.12) per 100,000 population. In the G20 aggregate, the number of deaths decreased by 22.0%, from 4,724.83 (95% UI, 3,445.46–6,286.86) to 3,683.62 (95% UI, 2,798.08–5,033.79), while the age-standardized death rate decreased by 55.5%, from 0.23 (95% UI, 0.17–0.31) to 0.10 (95% UI, 0.08–0.14) per 100,000 population.

Joinpoint regression analysis further characterized the overall temporal patterns in ASRs (Figure 1B). In China, significant decreases were observed in the age-standardized rates of DALYs, deaths, and YLLs, with AAPCs of −3.93% (95% CI, −4.26% to −3.61%), −3.96% (95% CI, −4.42% to −3.49%), and −4.08% (95% CI, −4.42% to −3.74%), respectively. The age-standardized prevalence rate and age-standardized YLD rate increased significantly, with AAPCs of 0.16% (95% CI, 0.11% to 0.22%) and 0.17% (95% CI, 0.11% to 0.22%), respectively, whereas the trend in the age-standardized incidence rate was not statistically significant (AAPC, −0.02%; 95% CI, −0.04% to 0.01%).

In the G20 aggregate, the age-standardized rates of DALYs, deaths, and YLLs also decreased significantly, with AAPCs of −2.36% (95% CI, −2.67% to −2.05%), −2.42% (95% CI, −2.76% to −2.08%), and −2.44% (95% CI, −2.76% to −2.12%), respectively. The age-standardized incidence rate increased significantly (AAPC, 0.12%; 95% CI, 0.09% to 0.15%), whereas the trends in the age-standardized prevalence rate and age-standardized YLD rate were not statistically significant, with identical AAPCs of 0.02% (95% CI, −0.02% to 0.05%).

### Sex-specific patterns in the burden of pulmonary arterial hypertension

**Figure 2.**
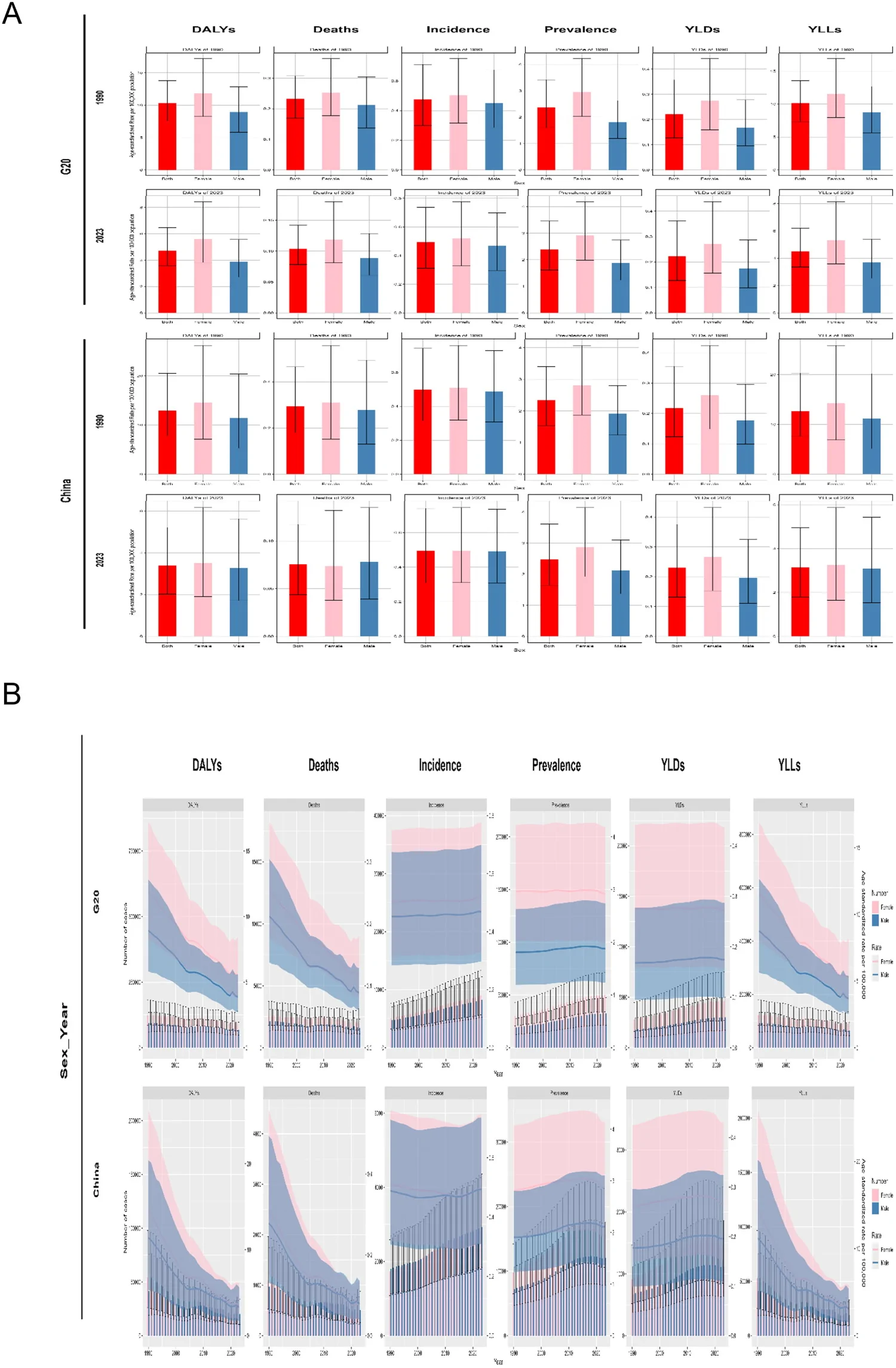
Sex-specific burden of pulmonary arterial hypertension in China and the G20 aggregate, 1990–2023. (A) Absolute numbers and age-standardized rates by sex in 1990 and 2023. Error bars indicate 95% uncertainty intervals. (B) Temporal changes in age-standardized rates and absolute numbers by sex during the study period.

Sex-specific patterns in the burden of PAH differed between China and the G20 aggregate (Figure 2A). In 2023, females had higher age-standardized prevalence and YLD rates than males in both settings. In China, the age-standardized prevalence rate was 2.87 (95% UI, 1.92–4.14) per 100,000 population among females and 2.11 (95% UI, 1.37–3.10) among males. The corresponding rates in the G20 aggregate were 2.92 (95% UI, 1.98–4.19) and 1.88 (95% UI, 1.23–2.74) per 100,000 population, respectively.

For mortality-related outcomes, higher rates among females were more evident in the G20 aggregate than in China. In 2023, the age-standardized DALY rate in the G20 aggregate was 5.60 (95% UI, 3.83–8.44) per 100,000 population among females, compared with 3.87 (95% UI, 2.71–5.57) among males. The corresponding age-standardized death rates were 0.12 (95% UI, 0.08–0.18) and 0.09 (95% UI, 0.06–0.13) per 100,000 population. In China, however, the female and male age-standardized DALY rates were closer, at 3.51 (95% UI, 1.90–6.17) and 3.28 (95% UI, 1.71–5.63) per 100,000 population, respectively, while the age-standardized death rate was slightly lower among females than males.

Temporal patterns also varied by setting (Figure 2B). In the G20 aggregate, female age-standardized rates were generally higher than male rates across the six measures during most of the study period. In China, the female-male differences in DALYs, deaths, and YLLs narrowed substantially between 1990 and 2023, whereas female rates of prevalence and YLDs remained higher than male rates.

### Age-specific patterns in the burden of pulmonary arterial hypertension

**Figure 3.**
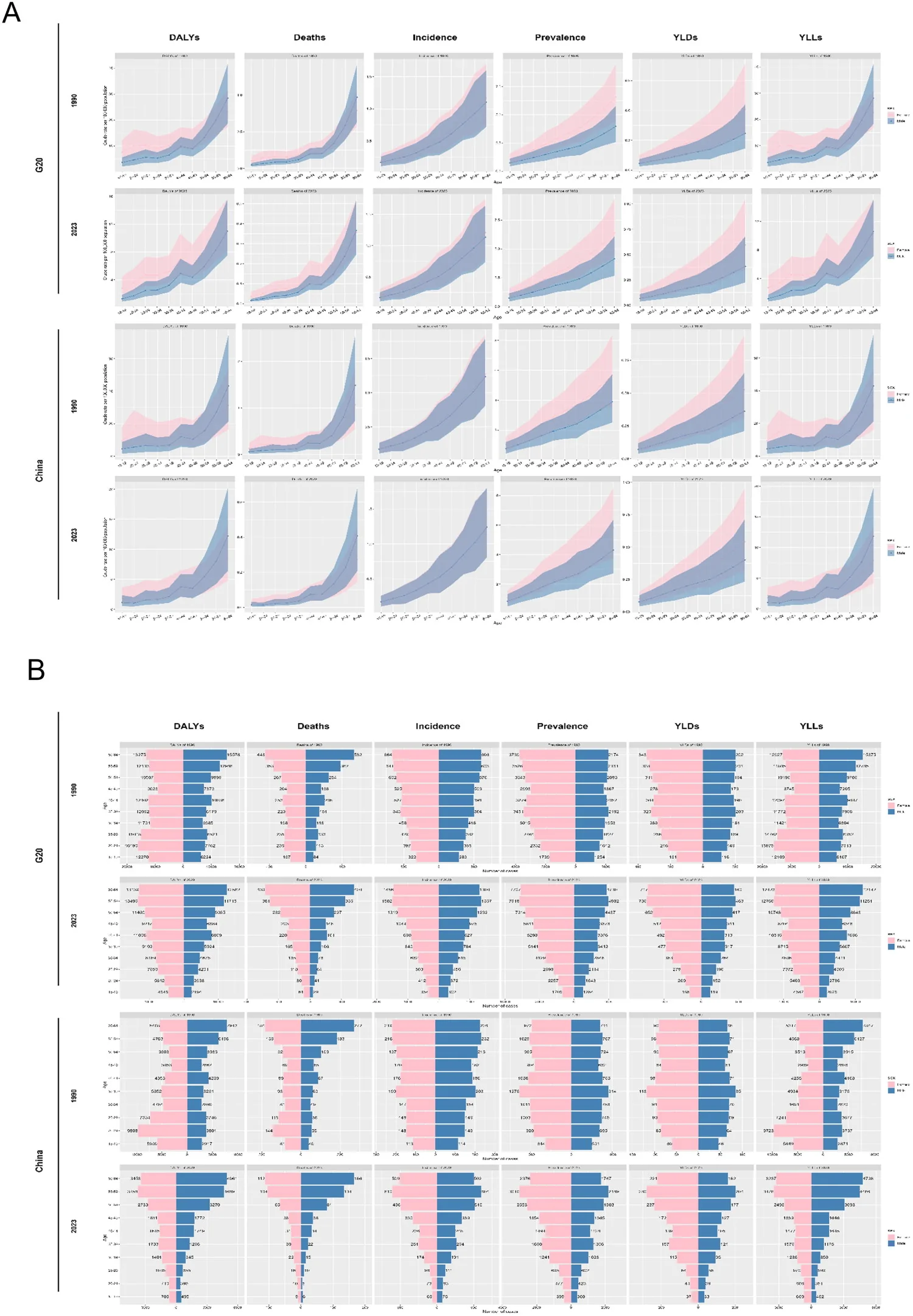
Age-specific patterns and changes in the distribution of pulmonary arterial hypertension burden in China and the G20 aggregate, 1990–2023. (A) Age-specific rates of incidence, prevalence, deaths, DALYs, YLDs, and YLLs in 1990 and 2023. (B) Absolute numbers of these outcomes across age groups in 1990 and 2023.

The age-specific burden of PAH varied substantially across the age groups analyzed in both China and the G20 aggregate (Figure 3A). In general, age-specific rates increased with age and were highest in the older analyzed age groups. Mortality-related measures, including death, DALY, and YLL rates, were lower in 2023 than in 1990 across most age groups. In China, the death rate among individuals aged 60–64 years decreased from 1.29 (95% UI, 0.81–2.06) per 100,000 population in 1990 to 0.34 (95% UI, 0.20–0.51) in 2023. The corresponding rate in the G20 aggregate decreased from 0.88 (95% UI, 0.65–1.14) to 0.36 (95% UI, 0.27–0.49) per 100,000 population.

Age-specific incidence, prevalence, and YLD rates showed comparatively limited changes over the study period. In China, the prevalence rate among individuals aged 60–64 years increased from 4.75 (95% UI, 3.03–6.95) per 100,000 population in 1990 to 5.06 (95% UI, 3.23–7.43) in 2023, whereas the incidence rate remained broadly stable at 1.25 and 1.24 per 100,000 population, respectively. In the G20 aggregate, prevalence and YLD rates among individuals aged 60–64 years also remained broadly stable between 1990 and 2023.

The age distribution of the absolute PAH burden changed between 1990 and 2023 (Figure 3B). In China, DALYs, prevalent cases, and YLDs became increasingly concentrated in older age groups. A broadly similar pattern was observed in the G20 aggregate, although the age distributions differed across burden measures. Overall, the absolute PAH burden shifted toward older age groups over the study period.

### Drivers of change: demographic decomposition

**Figure 4.**
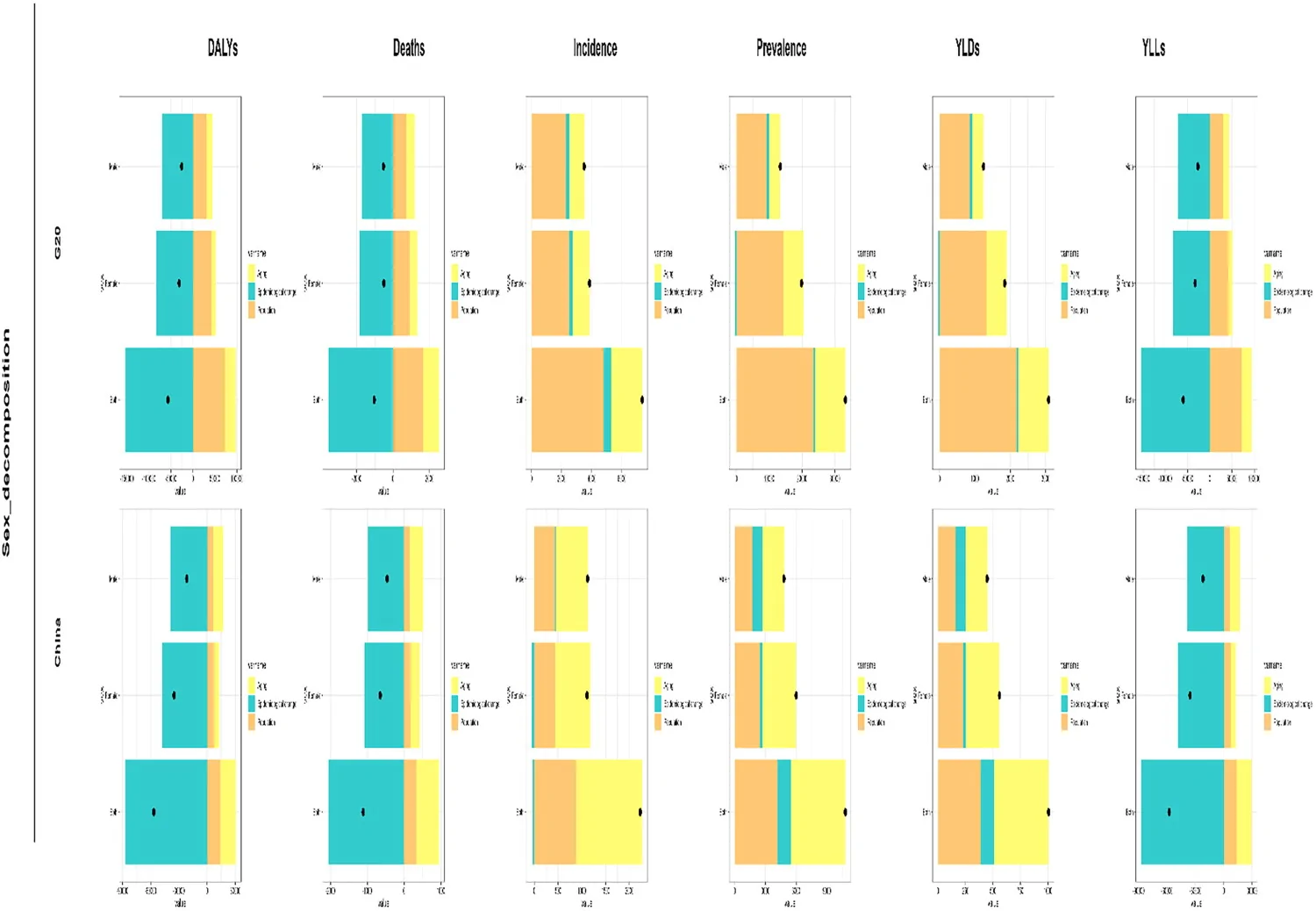
Decomposition of changes in the absolute burden of pulmonary arterial hypertension in China and the G20 aggregate, 1990–2023.

Decomposition analysis was used to quantify the contributions of population growth, population aging, and epidemiological change to changes in the absolute PAH burden between 1990 and 2023 (Figure 4). In China, the epidemiological-change component contributed to a reduction in the number of deaths, whereas population aging and population growth contributed in the opposite direction. Similar opposing contributions were observed for DALYs and YLLs. Population aging and population growth generally contributed to increases in nonfatal burden indicators in both China and the G20 aggregate. The epidemiological-change component represents the residual change remaining after accounting for population size and age structure and should not be interpreted as a direct measure of treatment effects, clinical improvement, or true changes in disease occurrence.

### Future projections of the burden of PAH

**Figure 5.**
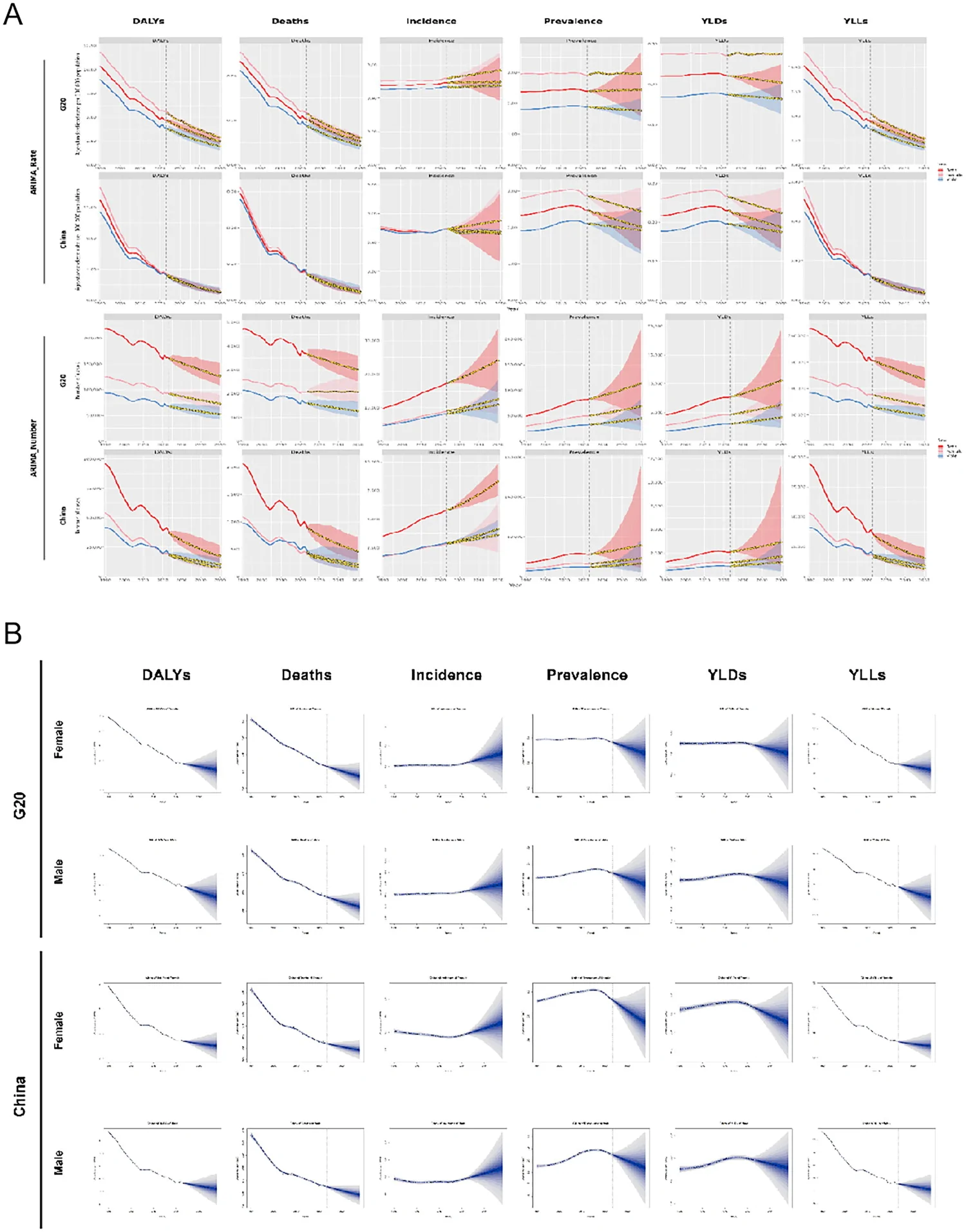
Projected burden of pulmonary arterial hypertension in China and the G20 aggregate. (A) ARIMA projections of absolute numbers and age-standardized rates for 2024–2050. Historical estimates are shown for 1990–2023, and shaded areas indicate 95% bootstrap prediction intervals; the dashed line marks the start of projection.(B) Sex-specific BAPC projections of age-standardized rates for 2024–2038 in females and males. Shaded areas indicate 95% Bayesian credible intervals.

Future projections suggested divergent trajectories between fatal and non-fatal burden indicators in both China and the G20 aggregate (Figure 5). Under the fitted ARIMA models, age-standardized DALY, death, and YLL rates were projected to decline continuously through 2050, whereas age-standardized incidence, prevalence, and YLD rates were projected to remain broadly stable. Conversely, the absolute numbers of incident and prevalent cases are expected to increase over the projection horizon.

Sex-specific BAPC projections through 2038 indicated that historical sex disparities will largely persist. Projected age-standardized DALY and prevalence rates remained higher among females than males in both settings, while the direction and magnitude of sex differences in age-standardized death rates varied between China and the G20 aggregate.

## Discussion

Using GBD 2023 data, this comparative analysis characterises 34-year trends and structural changes in the burden of pulmonary arterial hypertension (PAH) in China and the G20 aggregate. We observed a clear divergence at the population level: age-standardised mortality-related indicators declined substantially, whereas absolute case numbers and non-fatal burden continued to increase. Demographic decomposition analysis indicated that the expanding patient population was driven primarily by population growth and population ageing rather than increases in age-specific disease occurrence. These findings suggest an epidemiological transition in the population burden of PAH. Once associated with rapid mortality, PAH has increasingly become a chronic condition affecting an ageing population, creating growing demands for long-term surveillance and healthcare services.

Sustained declines in mortality-related indicators represented the most consistent trend across both study populations. In China, total PAH deaths decreased by 53.9%, accompanied by a 74.3% reduction in the age-standardised death rate, while similar downward trends were observed in the G20 aggregate. Our findings are consistent with previous GBD-based studies reporting long-term reductions in age-standardised mortality and years of life lost (YLL) associated with PAH [10,11]. Over the past three decades, improvements in diagnosis, risk stratification, specialist care, and access to PAH-targeted therapies may collectively have contributed to these population-level mortality improvements [12,13]. However, GBD data do not contain individual-level information on treatment, diagnostic timing, disease severity, or healthcare utilisation. Consequently, these population-level trends should not be interpreted as evidence for the effectiveness of any specific treatment, diagnostic strategy, or healthcare policy.

The increase in absolute case numbers should be interpreted separately from changes in age-standardized rates. Prevalent cases increased by 63.5% in China and by 67.3% in the G20 aggregate, while age-standardized prevalence changed only slightly. A similar separation between rising absolute prevalence and relatively stable age-standardized prevalence has been reported in previous GBD analyses of PAH [14].In the present study, this pattern was consistent with an important contribution from population growth and changes in age structure. Other factors, including changes in disease recognition, case ascertainment, and survival, may also influence prevalence estimates [15,16], but their individual contributions could not be quantified using the available data.

Sex differences in PAH burden varied across regions and outcome measures. In the G20 aggregate, females also had higher mortality-related rates, whereas the corresponding female-male differences were smaller in China, and the age-standardized death rate was slightly lower among females. These results are compatible with the recognized female predominance in PAH prevalence, while also showing that sex differences in fatal burden are not uniform across populations or outcomes [17]. Biological and clinical mechanisms underlying sex differences in PAH have been proposed [18], but these mechanisms were not assessed in the present study.

Age-specific analyses showed that PAH burden generally increased with age, with the greatest burden observed in the oldest age groups analyzed. The age groups contributing the largest absolute numbers also shifted toward older ages between 1990 and 2023, particularly in China. Previous studies have similarly reported a greater PAH burden among older populations [19]. In the present analysis, however, the shift in age distribution should primarily be interpreted in relation to population aging and changes in population structure. The available estimates did not include individual-level information on comorbidity, diagnostic delay, functional status, or treatment tolerability; these factors therefore should not be inferred directly from the observed age patterns.

The decomposition analysis helps clarify why trends in absolute numbers differed from trends in age-standardized rates. Population growth and population aging contributed positively to changes in absolute burden, whereas the epidemiological-change component reduced deaths, DALYs, and YLLs. The epidemiological-change component represents the change remaining after accounting for population size and age structure; it should not be interpreted as a direct measure of treatment effect or as evidence of ‘true epidemiological improvement.’ In addition, the reported components are counterfactual contributions to the net change rather than mutually exclusive proportions of a common total, and they are therefore not expected to sum to 100%. This interpretation avoids assigning the epidemiological component to specific clinical or policy mechanisms that were not measured.

The projection analyses suggested that the divergence between fatal burden and case volume may continue through 2050. Under the fitted ARIMA models, age-standardized death, DALY, and YLL rates were projected to decline, whereas age-standardized incidence, prevalence, and YLD rates were projected to remain broadly stable or change only slightly. At the same time, the absolute numbers of incident and prevalent cases were projected to increase. These projections extend historical patterns and should not be interpreted as deterministic forecasts. Future changes in diagnostic criteria, data availability, treatment access, population structure, and healthcare policy could lead to trajectories that differ from those estimated here [20]^.^ The projected increase in prevalent cases may nevertheless have implications for the volume of long-term follow-up and specialist assessment required, without establishing the magnitude of future healthcare demand.

This study has several strengths. It used the updated GBD 2023 estimates and evaluated six complementary outcomes using both absolute numbers and age-standardized rates. Joinpoint regression was used to characterize changes in temporal trends, demographic decomposition separated broad population and epidemiological contributions, and ARIMA and BAPC models were used to examine possible future trajectories. Sex- and age-specific analyses provided additional detail beyond the overall China-versus-G20-aggregate comparison. Together, these approaches provide a multidimensional description of PAH burden, but they do not establish causal relationships. Several limitations should be considered when interpreting our findings. First, the GBD 2023 estimates are derived from multiple data sources and standardized modelling procedures; therefore, the results are most appropriately interpreted at the population level. Second, although the GBD cause category used in this study was defined as pulmonary arterial hypertension (cause ID 494), the available estimates did not allow further stratification by detailed clinical phenotype, underlying etiology, or WHO pulmonary hypertension subgroup. Third, the G20 estimates were analysed as an IHME-provided supranational aggregate and may therefore not fully reflect heterogeneity across individual member countries. Fourth, the epidemiological-change component in the decomposition analysis reflects changes in age-specific rates after accounting for population size and age structure and should be interpreted as a composite population-level effect rather than a direct measure of treatment efficacy. Finally, both ARIMA and BAPC projections were based on the continuation of historical patterns and were subject to increasing uncertainty over extended projection horizons. The ARIMA models projected trends through 2050, whereas the BAPC projection horizon extended through 2038. Given the increasing uncertainty associated with longer-term extrapolation, these projections should be interpreted as indicative trend scenarios rather than precise estimates of future burden.

## Conclusions

From 1990 to 2023, mortality-related indicators of pulmonary arterial hypertension declined in China and the G20 aggregate, whereas the absolute numbers of incident and prevalent cases increased. Population growth and population aging contributed to the divergence between absolute and age-standardized trends. Sex- and age-specific patterns varied across outcomes and between China and the G20 aggregate. Model-based projections suggested that declining mortality-related burden may coexist with a continued increase in the number of people living with PAH. These findings may inform future PAH surveillance and long-term health-service planning, although the projections should be interpreted cautiously.

## Data Availability

The GBD datasets analyzed herein are fully open to the public with no embargo or access fees. All researchers can obtain the data freely through the official GHDx platform at http://ghdx.healthdata.org/gbd-results-tool. Only a free, non-restrictive individual registration is required by the platform itself.

## Abbreviations

PAH: Pulmonary arterial hypertension
G20: Group of Twenty
DALY: Disability adjusted life year
EAPC: Estimated annual percentage change
GBD: Global Burden of Disease
ASR: Age standardized rate
CI: Confidence interval
YLD: Year lived with disability
YLL: Year of life lost
ARIMA: Autoregressive integrated moving average
BAPC: Bayesian age–period–cohort
AAPC: Average annual percentage change
APC: Annual percentage change
ASIR: Age-standardized incidence rate
ASPR: Age-standardized prevalence rate
ASDR: Age-standardized death rate
UI: Uncertainty interval
IHME: Institute for Health Metrics and Evaluation

## References

[1] Humbert M, Kovacs G, Hoeper MM, et al. ERS Scientific Document Group. Eur Respir J. 2023;61:2200879–36028254.36028254

[2] Huang H, Hu C, Zhang R, et al. Global burden of pulmonary arterial hypertension and associated heart failure: global burden of disease 2021 analysis. JACC Heart Fail. 2025;13(10):102385. Epub 2025 Mar 5. Erratum in: JACC Heart Fail. 2025 May;13(5):886. doi:10.1016/j.jchf.2024.12.005. 40047764

[3] Ramani G, Bali V, Black H, et al. Exploring the economic burden of pulmonary arterial hypertension and its relation to disease severity and treatment escalation: a systematic literature review. Pharmacoeconomics. 2025;43(7):741–760. doi:10.1007/s40273-025-01492-1. 40244370 PMC12167309

[4] GBD 2023 Disease and Injury and Risk Factor Collaborators. Burden of 375 diseases and injuries, risk-attributable burden of 88 risk factors, and healthy life expectancy in 204 countries and territories, including 660 subnational locations, 1990–2023: a systematic analysis for the Global Burden of Disease Study 2023. Lancet. 2025;406(10513):1873–1922. doi:10.1016/S0140-6736(25)01637-X. 41092926 PMC12535840

[5] GBD 2023 Cancer Collaborators. The global, regional, and national burden of cancer, 1990–2023. Lancet. 2025;406(10512):1565–1586. doi:10.1016/S0140-6736(25)01635-6. 41015051 PMC12687902

[6] Devleesschauwer B, Haagsma JA, Angulo FJ, et al. Methodological framework for world health organization estimates of the global burden of foodborne disease. PLoS One. 2015;10(12):e0142498. doi:10.1371/journal.pone.0142498. 26633883 PMC4668830

[7] Wu X, Suo S, Su X, et al. Trends in pulmonary arterial hypertension: insights from Global Burden of Disease 1990–2021. BMJ Open. 2025;15(3):e095348. doi:10.1136/bmjopen-2024-095348. PMC11927429 40107705

[8] Leber L, Beaudet A, Muller A. Epidemiology of pulmonary arterial hypertension and chronic thromboembolic pulmonary hypertension: identification of the most accurate estimates from a systematic literature review. Pulm Circ. 2021;11(1):2045894020977300–2045894020977312. doi:10.1177/2045894020977300. 33456755 PMC7797595

[9] Chen L, Wu X, Gao X. Trends and cross-country disparity in the burden of pulmonary arterial hypertension among women of childbearing age from 1990 to 2021. Front Glob Womens Health. 2025;6:1651601. doi:10.3389/fgwh.2025.1651601. 41756755 PMC12932459

[10] Liu Z, Mo L, Cao W, et al. Global, regional, and national trends in pulmonary arterial hypertension burden, 1990–2021: findings from the global burden of disease study 2021. Front Public Health. 2025;13:1516365. doi:10.3389/fpubh.2025.1516365. 40510578 PMC12158705

[11] Huang S, Qiu J, Wang A, et al. Burden of pulmonary arterial hypertension in Asia from 1990 to 2021: findings from Global Burden of Disease Study 2021. Chin Med J (Engl). 2025;138(11):1324–1333. doi:10.1097/CM9.0000000000003559. 40375470 PMC12150979

[12] Hoeper MM, Pausch C, Grünig E, et al. Temporal trends in pulmonary arterial hypertension: results from the COMPERA registry. Eur Respir J. 2022;59(6):2102024. doi:10.1183/13993003.02024-2021. 34675047 PMC9160392

[13] Appenzeller P, Lichtblau M, Berlier C, et al. Disease characteristics and clinical outcome over two decades from the Swiss pulmonary hypertension registry. Pulm Circ. 2022;12(1):e12001. doi:10.1002/pul2.12001. 35506112 PMC9052988

[14] Guan A, Dai Z, Lin J, et al. Global disease burden of pulmonary arterial hypertension: patterns and inequality in 1990–2021, with projections to 2035. BMC Public Health. 2026;26(1):4. doi:10.1186/s12889-025-24972-7. 41484573 PMC12763842

[15] Morland K, Gerges C, Elwing J, et al. Real-world evidence to advance knowledge in pulmonary hypertension: status, challenges, and opportunities. A consensus statement from the Pulmonary Vascular Research Institute’s Innovative Drug Development Initiative’s Real-world Evidence Working Group. Pulm Circ. 2023;13(4):e12317. doi:10.1002/pul2.12317. 38144948 PMC10739115

[16] Emmons-Bell S, Johnson C, Boon-Dooley A, et al. Prevalence, incidence, and survival of pulmonary arterial hypertension: a systematic review for the global burden of disease 2020 study. Pulm Circ. 2022;12(1):e12020. doi:10.1002/pul2.12020. 35506069 PMC9052982

[17] Cheron C, McBride SA, Antigny F, et al. Sex and gender in pulmonary arterial hypertension. Eur Respir Rev. 2021;30(162):200330. doi:10.1183/16000617.0330-2020. 34750113 PMC9488668

[18] Bousseau S, Gu S, Lahm T. Biological and clinical implications of sex differences in right heart failure associated with pulmonary arterial hypertension. Arterioscler Thromb Vasc Biol. 2026;46(6):e322096. doi:10.1161/ATVBAHA.126.322096. 42059076

[19] Zhou L, Luo J, Tan C, et al. Worldwide burden of pulmonary arterial hypertension in adults aged 65 years and older from 1990 to 2021. J Am Geriatr Soc. 2025;73(12):3700–3707. doi:10.1111/jgs.70135. 41017022

[20] Cheng Y, Lin Y, Shi H, et al. Projections of the Stroke Burden at the Global, Regional, and National Levels up to 2050 Based on the Global Burden of Disease Study 2021. J Am Heart Assoc. 2024;13(23):e036142. doi:10.1161/JAHA.124.036142. 39575720 PMC11681572

